# Successful C1 inhibitor short-term prophylaxis during redo mitral valve replacement in a patient with hereditary angioedema

**DOI:** 10.1186/1749-8090-5-86

**Published:** 2010-10-18

**Authors:** Jonathan A Bernstein, Suzanne Coleman, Arturo J Bonnin

**Affiliations:** 1 University of Cincinnati, Department of Internal Medicine, Division of Immunology/Allergy Section, 231 Albert Sabin Way, Cincinnati, Ohio, USA; 2 Innovation Center, Kettering Health Network, 3535 Southern Blvd, Kettering, Ohio, USA; 3 Allergy and Asthma Centre of Dayton, 8039 Washington Drive, Suite 100, Centerville, Ohio, USA

## Abstract

Hereditary angioedema is characterized by sudden episodes of nonpitting edema that cause discomfort and pain. Typically the extremities, genitalia, trunk, gastrointestinal tract, face, and larynx are affected by attacks of swelling. Laryngeal swelling carries significant risk for asphyxiation. The disease results from mutations in the C1 esterase inhibitor gene that cause C1 esterase inhibitor deficiency. Attacks of hereditary angioedema result from contact, complement, and fibrinolytic plasma cascade activation, where C1 esterase inhibitor irreversibly binds substrates. Patients with hereditary angioedema cannot replenish C1 esterase inhibitor levels on pace with its binding. When C1 esterase inhibitor is depleted in these patients, vasoactive plasma cascade products cause swelling attacks. Trauma is a known trigger for hereditary angioedema attacks, and patients have been denied surgical procedures because of this risk. However, uncomplicated surgeries have been reported. Appropriate prophylaxis can reduce peri-operative morbidity in these patients, despite proteolytic cascade and complement activation during surgical trauma. We report a case of successful short-term prophylaxis with C1 esterase inhibitor in a 51-year-old man with hereditary angioedema who underwent redo mitral valve reconstructive surgery.

## Background

Attacks of hereditary angioedema (HAE) are characterized by sudden episodes of brawny, nonpitting edema, causing discomfort and pain[1]. Areas of the body typically affected include the extremities, genitalia, trunk, gastrointestinal tract, face, and larynx. Untreated patients with HAE are at risk for deadly attacks of laryngeal swelling, where up to 30% may asphyxiate[2].

HAE is an inherited autosomal dominant disorder resulting from any number of mutations in the C1 esterase inhibitor (C1 INH) gene that cause C1 INH deficiency[2]. Approximately 85% of cases are type 1 HAE, which is characterized by reduced levels of circulating C1 INH[3,4]. The remaining 15% of cases are type 2 HAE, which is characterized by dysfunctional circulating C1 INH[3,4]. A child will have a 50% chance of inheriting HAE if one parent has the disease; however, 25% of cases arise from de novo mutations[3]. Inherited angioedema with normal C1 inhibitor levels has been described and is thought to be a separate disease resulting from a factor XII missense mutation that leads to bradykinin overproduction[5,6].

**Figure 1:**
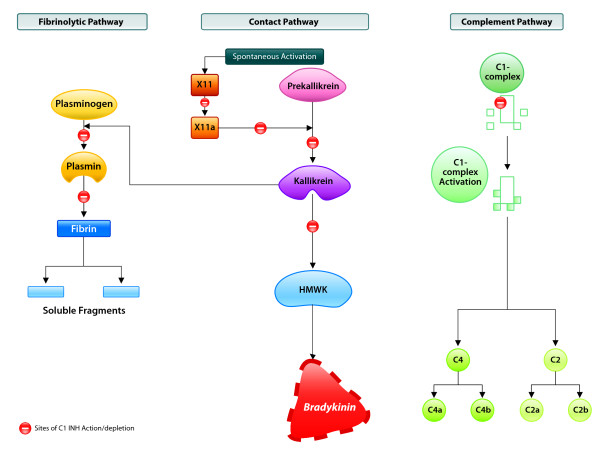
**C1 INH action in plasma cascades**. Depletion of C1 INH due to plasma cascade activation allows bradykinin overproduction in patients with hereditary angioedema. Bradykinin mediates acute swelling in these patients. Reproduced with permission from Weis[15].

Histamine mediated allergic inflammation is not involved in HAE[7]. Instead, HAE attacks result from contact, complement, and fibrinolytic plasma cascade activation, where C1 INH is a suicide inhibitor[2]. People with HAE have defective C1 INH synthesis with typical C1 INH levels that are 5%-30% of normal.^2 ^Bradykinin is generated in large quantities via the contact pathway once C1 INH is depleted (Figure 1)[2]. Excess bradykinin production leads to acute HAE attacks as a result of increased vasodilatation, vascular permeability, and contraction of nonvascular smooth muscle[3].

HAE affects 1:50,000 people[8]. Fifty percent of patients will develop symptoms by age 10, although attacks have been reported in children as young as 2 years old[9]. Symptom frequency and severity may be extremely variable, even within families[4]. There may be no obvious trigger for attacks and no correlation between attack severity and subtype of disease. However, local trauma, stress, and hormonal fluctuations in women may be responsible for many attacks[10].

Despite the inherent risks of performing surgery on patients with HAE, the cardiovascular surgery literature provides examples of uncomplicated surgery in patients who were methodically prophylaxed with different agents[11,12]. Appropriate choice of a prophylactic agent and its judicious use can help surgeons reduce peri-operative morbidity to patients, despite multiple sources of proteolytic cascade and complement activation known to occur with surgical trauma, and more specifically, with cardiac pump bypass surgeries[13,14].

Previous agents used for prophylactic treatment of HAE patients undergoing surgery include fresh frozen plasma, high-dose attenuated androgens, and anti-fibrinolytic agents. No agent is currently approved for short-term procedural prophylaxis. However, newer agents are approved for long-term prophylaxis (C1 esterase inhibitor [CINRYZE™]) and acute attacks (C1 esterase inhibitor [Berinert P^®^] and kallikrein inhibitor, ecallantide [KALBITOR^®^]). Here we report a case of successful short-term prophylaxis using C1 INH in a 51-year-old man with HAE undergoing redo mitral valve reconstructive surgery.

## Case presentation

A 51-year-old man with type 2 HAE, a history of acute respiratory failure, chronic airway obstruction, adhesive pericarditis, congestive heart failure, chronic pulmonary heart disease, and previous mitral valve annuplasty was scheduled for redo surgery six months after the initial surgical procedure due to severe mitral valve regurgitation. During the initial surgical procedure, the patient was successfully prophylaxed with C1 INH and his peri-operative course was uneventful. Details of the initial surgery were previously published in abstract form[12].

The patient reported onset of HAE symptoms beginning at age 12. Previous attacks consisted of abdominal, facial, extremity, and painful genital swelling. He reported two previous episodes of severe laryngeal edema secondary to oral surgeries. The patient was controlled on long-term prophylaxis with the attenuated androgen, danazol 400 mg daily, under the care of an allergist. However, even while on this medication, he reported breakthrough swelling including laryngeal edema. Historically, his C1 INH levels were normal, but the C1 INH was dysfunctional, and he had a persistently low C4 level - typical findings in type 2 HAE.

Two months after the initial mitral valve reconstruction and annuloplasty surgery, the patient presented to the emergency department in heart failure due to pericardial effusion. The patient's surgeon and allergist were consulted prior to any procedure. As a result, the patient was prophylaxed with C1 INH 1000 units prior to pericardiocentesis, where one liter of pericardial fluid was removed. A transesophageal echocardiogram showed moderated-to-severe mitral valve regurgitation secondary to failure of previous repair. The procedure was tolerated without swelling.

The patient was scheduled for redo mitral valve repair six months later. Laboratory studies prior to surgery preparations were unremarkable except for chronic anemia. Cardiac catheterization was performed 48 hours prior to repeat mitral valve reconstruction. C1 INH 1000 units was administered six hours before cardiac catheterization. Since the patient was on danazol 400 mg daily, his functional C1 INH levels were within the normal range, but his C4 level remained low. C1 INH 1000 units was again administered intravenously twelve hours before repeat mitral valve reconstruction. During the redo surgery, the patient underwent sternotomy with lysis of extensive mediastinal adhesions, redo mitral valve repair with resection of chordae tendineae, and closure of dehisced prior leaflet closure; removal of annuloplasty band and insertion of 32 CardioMedics annuloplasty ring; and intraoperative 2-D esophageal echocardiogram for aortic ultrasound.

The surgery was uncomplicated without excessive blood loss. The patient experienced no postoperative complications associated with the surgery or HAE. He was extubated, the Swan-Ganz was discontinued, and danazol 400 mg daily prophylaxis was resumed on postoperative day one. Chest tubes were removed on postoperative day two, and the patient was transferred to the regular nursing floor. He was discharged on postoperative day 4 and instructed to follow-up with the allergist caring for his HAE as an outpatient.

## Conclusions

Three medications are available for acute treatment of HAE: Berinert-P^® ^(plasma-derived C1 esterase inhibitor, approved in the US and Europe), KALBITOR^® ^(ecallantide, a kallikrein inhibitor, approved only in the US currently), and Firazyr^® ^(icatibant, bradykinin β2-receptor inhibitor, approved only in Europe currently). Two medications are available for long-term prophylaxis: CINRYZE™ (C1 esterase inhibitor [human], approved in the US) and the attenuated androgen, danazol (approved in the US and Europe). The newer medications target the underlying pathology of HAE and have less potential for long-term side effects than androgens. No medication is currently approved for procedural prophylaxis. However, consensus guidelines recommend the use of C1 INH (Table 1) and there are now many case reports of using C1 INH pre-operatively in Europe and the US [8,12].

**Table 1:** Consensus guideline C1 INH dosages for procedural prophylaxis

Patient weight	C1 INH dose
≤50 kg	500 units

>50 kg to ≤100 kg	1000 units

>100 kg	1500 units

Administer 1 hour before procedure. Have additional doses available if needed during and after surgery. C1 INH = C1 esterase inhibitor.

This case demonstrates that major surgery can be performed on HAE patients if care is closely coordinated. One week after surgery, this patient's long-term danazol prophylaxis dosage was reduced to 200 mg once daily. With close management, this patient now experiences only a few breakthrough symptoms during normal activity, and he has needed procedural prophylaxis with C1 INH for a dental procedure only once since his mitral valve surgery. This case also demonstrates that typical long-term prophylactic doses of C1 INH may be sufficient for prophylaxis prior to major surgery. Prior to his initial mitral valve replacement, the patient received 2000 units of C1 INH. However, after consulting an HAE expert, the doses for subsequent procedures were reduced to 1000 units with comparable results.

Procedural prophylaxis protocols should include a thorough patient history, coordinated efforts among medical specialties, family education, and use of an appropriate prophylactic medication. The successful use of a procedural prophylaxis protocol in this case will hopefully encourage the use of appropriate prophylaxis in patients with HAE who may be otherwise denied surgery.

## Consent

Written informed consent was obtained from the patient for publication of this case report. A copy of the written consent is available for review by the Editor-in-Chief of this journal.

## Competing interests

The authors received honoraria for the development of this manuscript. SC has received research support from ViroPharma Incorporated. AB has received speaker fees from Merck, Genentech/Novartis, and GlaxoSmithKline.

## Authors' contributions

JB, SC, and AJB contributed to the initial concept and design of the manuscript, provided the data presented, performed critical review of the medical concepts, and read and approved the final version.
